# A Multimodality Hybrid Gamma-Optical Camera for Intraoperative Imaging

**DOI:** 10.3390/s17030554

**Published:** 2017-03-09

**Authors:** John E. Lees, Sarah L. Bugby, Mohammed S. Alqahtani, Layal K. Jambi, Numan S. Dawood, William R. McKnight, Aik H. Ng, Alan C. Perkins

**Affiliations:** 1 Space Research Centre, Michael Atiyah Building, University of Leicester, Leicester LE1 7RH, UK; s.bugby@le.ac.uk (S.L.B.); msma7@le.ac.uk (M.S.A.); lj97@le.ac.uk (L.K.J.); nsdd2@le.ac.uk (N.S.D.); wm68@le.ac.uk (W.R.M.); 2 Radiological Sciences, Division of Clinical Neuroscience, School of Medical, University of Nottingham, Nottingham NG7 2UH, UK; Aik.Ng@nottingham.ac.uk (A.H.N.); Alan.Perkins@nottingham.ac.uk (A.C.P.)

**Keywords:** optical imaging, NIR fluorescence, hybrid imaging, gamma camera, clinical images

## Abstract

The development of low profile gamma-ray detectors has encouraged the production of small field of view (SFOV) hand-held imaging devices for use at the patient bedside and in operating theatres. Early development of these SFOV cameras was focussed on a single modality—gamma ray imaging. Recently, a hybrid system—gamma plus optical imaging—has been developed. This combination of optical and gamma cameras enables high spatial resolution multi-modal imaging, giving a superimposed scintigraphic and optical image. Hybrid imaging offers new possibilities for assisting clinicians and surgeons in localising the site of uptake in procedures such as sentinel node detection. The hybrid camera concept can be extended to a multimodal detector design which can offer stereoscopic images, depth estimation of gamma-emitting sources, and simultaneous gamma and fluorescence imaging. Recent improvements to the hybrid camera have been used to produce dual-modality images in both laboratory simulations and in the clinic. Hybrid imaging of a patient who underwent thyroid scintigraphy is reported. In addition, we present data which shows that the hybrid camera concept can be extended to estimate the position and depth of radionuclide distribution within an object and also report the first combined gamma and Near-Infrared (NIR) fluorescence images.

## 1. Introduction

Gamma and multimodal imaging has heritage in large static imaging systems (Single-Photon Emission Computed Tomography (SPECT), Positron Emission Tomography (PET), SPECT-Computed Tomography (CT), PET-CT, PET-Magnetic Resonance (MR)). Recently, small field of view (SFOV) portable gamma imaging systems have been designed that could be taken into the operating room. Advances in gamma detection technology allow these to provide improved spatial resolution for specific procedures, such as sentinel node localisation, where this is of benefit.

Many research groups around the world have been developing a range of hand-held or portable systems for medical gamma imaging [1,2,3,4,5,6,7,8,9,10,11,12]. To date, these smaller systems have all concentrated on a single imaging modality. These have been evaluated for a range of clinical applications, however a major area of interest is in the localisation of sentinel nodes in patients with cancer. Sentinel Lymph Node Biopsy (SLNB) is the standard procedure for evaluating and staging a range of cancers—notably breast cancer, melanoma, and head and neck cancer. SLNB is an established, minimally invasive surgical technique commonly used to determine the likelihood of a cancer spreading from a primary tumour and metastasising throughout the lymphatic system. Current practice uses a radioisotope tracer, often in conjunction with a blue dye, to help the surgeon locate lymphatic ducts and sentinel lymph nodes [13]. Recently, there have been a number of studies that have shown that hybrid fluorescent-radioactive tracers, such as ICG-^99m^Tc-nanocolloid, improve the identification of sentinel lymph nodes when compared with the standard technique using blue dye [14,15]. However, this new approach still depends on non-imaging gamma detectors to locate deep-seated nodes.

This report describes recent developments that extend the hybrid concept to include an estimate of the position and depth of radionuclide distribution within an object and the first results of combined gamma and near-infrared (NIR) fluorescence imaging. A recent clinical image of a patient undergoing a thyroid scintigraphy investigation is also presented which shows the benefit of combined optical and gamma images.

## 2. Materials and Methods

### 2.1. Hybrid Gamma Camera

The Hybrid Gamma Camera (HGC) combines an optical and a gamma camera in a configuration that provides hybrid imaging with high spatial resolution and a variety of image formats as output. The HGC system is flexible and a number of collimators can be employed to match the spatial resolution and sensitivity requirements for various clinical applications.

The HGC has been designed to be sensitive over the energy range 30–140 keV. At present, the HGC uses a Hamamatsu CsI(Tl) columnar scintillator (Hamamatsu Photonics UK Ltd., Welwyn Garden City, UK) (1500 µm thick) directly coupled to a charge-coupled device (CCD) (e2v CCD97 BI, E2V Technologies Ltd., Chelmsford, UK) and a tungsten pinhole collimator. This thickness of scintillator has an absorption ranging from ~100% to 38% over the energy range of interest [12]. A simple, single pinhole collimator was chosen, partly to simplify the construction but also to extend the imaging field of view (FOV)—the field of view, when using a pinhole collimator, increases with the distance of the camera to the object. The collimators were manufactured (Ultimate Metals, London, UK) from tungsten and are 6 mm thick and 45 mm in diameter with two different pinhole diameters (0.5 mm or 1.0 mm diameter), each collimator having a 60° acceptance angle.

There are different constraints to consider in the selection of the pinhole collimators including pinhole diameter, collimator material, acceptance angle, and photon energy. The on-axis geometric sensitivities for 140 keV gamma photons were calculated to be 4.3 × 10^−5^ and 1.4 × 10^−4^ with 0.5 mm and 1.0 mm diameter pinholes, respectively.

The distance between the CCD and the pinhole is fixed at 10 mm. The magnification of the system depends on the distance between the object being imaged and the collimator. Gamma photons interact with the scintillator and generate optical photons, the number being related to the incident photon energy. The position and the energy of the incident photon are recorded by the hybrid system [12].

**Figure 1:**
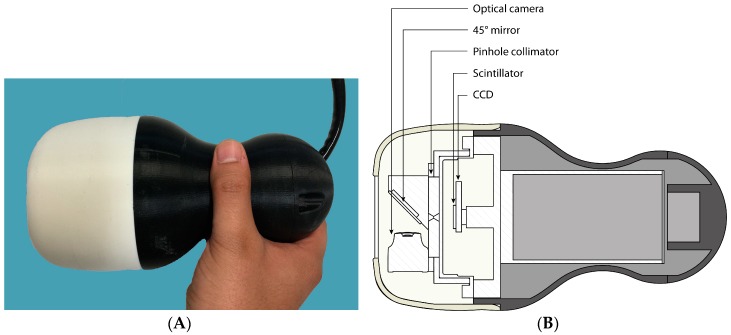
(**A**) Photograph of the Hybrid Gamma Camera (HGC); (**B**) schematic of the HGC showing the internal layout (for more details see Lees et al. [12]).

A thin (1 mm thick) first surface mirror at 45° to the collimator is positioned directly in front of the pinhole and reflects optical photons to an optical camera outside the direct line of sight of the pinhole [12]. Gamma photons from a source will pass through the mirror with minimal absorption (<1%) whereas optical photons will be reflected by the mirror towards the optical camera. The HGC (Figure 1) is connected to the imaging electronics system and controlled by a PC.

The HGC is shielded by tungsten (3 mm thick) and enclosed in a plastic enclosure for both electrical and thermal isolation. The HGC is displayed in Figure 1.

### 2.2. Near-Infrared (NIR) Fluorescence Imaging

Preliminary in vitro experiments were undertaken to demonstrate proof of principle that the system could be adapted for fluorescent imaging. The HGC was modified by the addition of a NIR enabled camera in place of the optical camera, with a separate LED ring as an excitation source (excitation wavelength 785 nm).

### 2.3. Phantoms

During the early development of the HGC, a number of phantoms were designed and manufactured to evaluate the performance of the system. These ranged from simple multi-well phantoms [16]—which investigated the spatial resolution of the gamma camera but also highlighted the difficulty in consistently filling very small holes (0.5 mm diameter) with a radioisotope solution—to more sophisticated designs that simulated sentinel lymph nodes and lymphatic vessels [17,18]. In addition, commercial phantoms which are widely available—such as the Picker Nuclear thyroid phantom (Part # 3602)—have been used to assess contrast-to-noise ratios and image enhancement techniques [19]. To further assess the HGC for clinical applications, two new phantoms were designed and manufactured: a breast phantom and a head and neck phantom.

### 2.4. Breast Phantom

**Figure 2:**
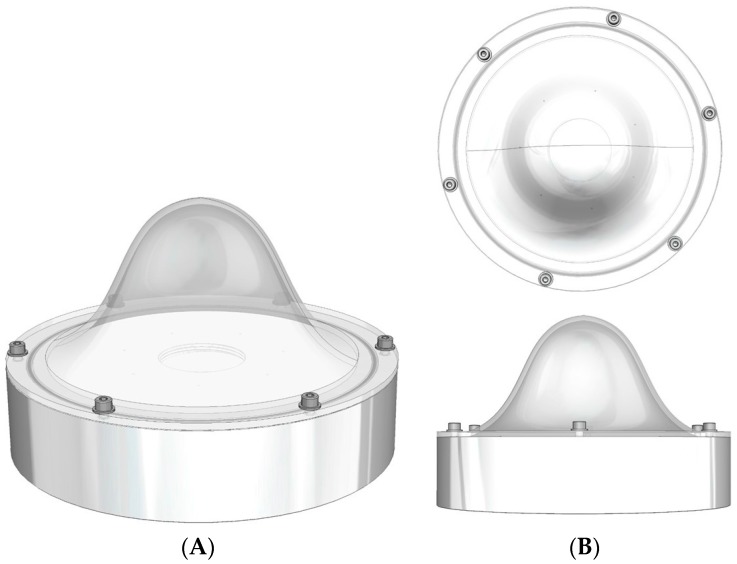
Schematic of the breast phantom. (**A**) An isometric view and; (**B**) a plan and side view.

The breast phantom was designed to assess the performance of the HGC in tumour and sentinel lymph node imaging. The aim was to produce a flexible phantom that could contain a number of objects of different sizes, each filled with a known amount of radioactivity, placed at a variety of positions within the phantom. In addition, the whole phantom could be filled with water or a diluted radioactive solution to mimic a general background activity level. Figure 2 shows a number of views of the phantom.

The dome has a diameter of 160 mm with a wall thickness of 3 mm, and sits on a base (base-to-dome apex height is 70 mm) of thickness 35 mm, inner diameter 160 mm and outer diameter 175 mm. A hole in the base section for filling the phantom is sealed using a simple plug and a rubber ring.

To minimise the manufacturing cost the phantom was fabricated from Perspex for the base and the filling plug and dome were made using a 3-D rapid prototyping processes.

**Figure 3:**
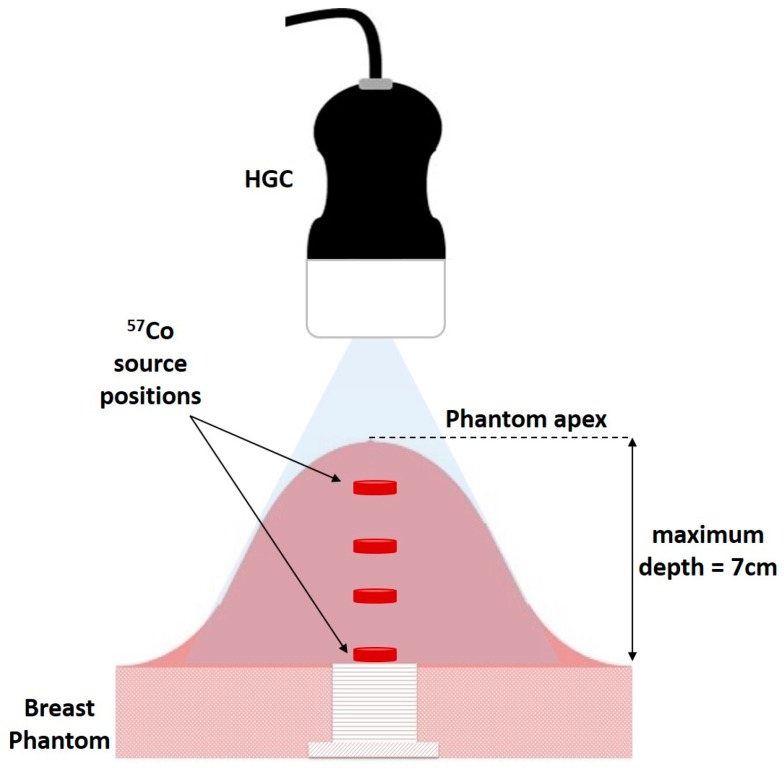
Schematic of experimental arrangement of HGC and breast phantom showing a source at different heights from apex.

A small ^57^Co capsule source (~8 mm diameter, activity ~90 MBq) was placed inside the phantom at a number of different positions (20, 33, 50, and 70 mm) below the apex of the dome, (Figure 3). The HGC was initially placed at 60 mm above the phantom at a camera viewing angle of 0°.

Both optical and gamma images were taken at this position, with the gamma images accumulating for 3 min. The HGC was then raised away from the phantom in steps of 20 mm to a maximum distance of 160 mm with images from both modalities taken at each vertical position.

### 2.5. Head and Neck Phantom

Lymphoscintigraphic and sentinel lymph node detection procedures for the head and neck present significant challenges for surgical teams due to the number of lymph nodes and their close proximity to one another and to surrounding vital structures. An intraoperative camera, with the ability to survey large areas of the surgical field while maintaining localisation, would be particularly beneficial in these procedures [9].

**Figure 4:**
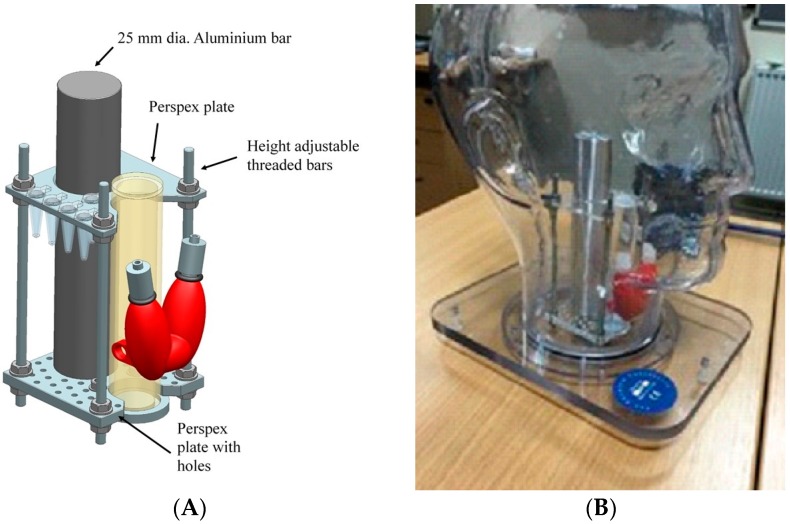
(**A**) Schematic of the insert for the head and neck phantom showing the thyroid, simplified trachea, and spine parts; (**B**) photograph of the complete phantom with the insert in position inside the head.

To evaluate the HGC for head and neck procedures, a phantom was designed which included a life-size thyroid, and simplified trachea and spine components. The design also included a jig to hold simulated lymph nodes, injection sites and primary tumours. Figure 4 shows the engineering design which was fabricated in-house with the full details to be reported elsewhere. The new insert was used in conjunction with a commercial phantom (The Phantom Laboratory, Salem, MA, USA)—the outer shell of the anthropomorphic head and neck phantom is water fillable and is constructed of cellulose acetate butyrate.

### 2.6. Near-Infrared Experiment

In a simple experiment an ^111^In-CW800 dendrimer probe with gamma emission energies of 254 keV and 171 keV and fluorescent dye emission wavelength of 794 nm was placed in an Eppendorf container and positioned in front of the modified HGC system. Experiments were carried out in a laboratory with overhead lights turned off. Images with both the gamma and fluorescent wavelengths were obtained and presented in a fused display.

## 3. Results

### 3.1. In Vitro Phantom Imaging

The three new phantoms have been used in a variety of imaging studies. A selection of results from these are highlighted below.

#### 3.1.1. Depth Estimation Using the Breast Phantom

One of the aims for the breast phantom was to use it as part of an assessment of depth estimation using the multimodality imaging capability of the HGC [20].

**Figure 5:**
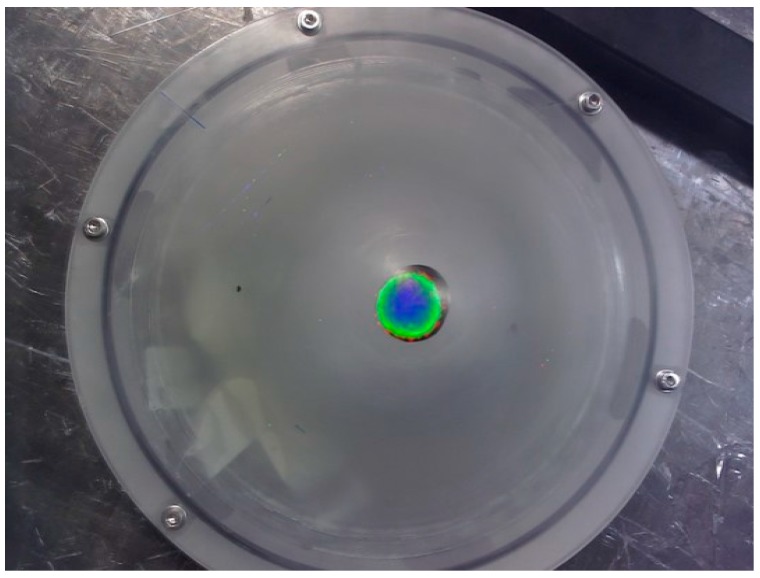
A fused optical and gamma image of a radioisotope source inside the breast phantom positioned at a distance of 70 mm below the apex.

Figure 5 shows a typical fused optical and gamma image at a height of 120 mm above the apex of the breast phantom. From this figure, it is clear that having the radioisotope distribution mapped directly onto the anatomy provides additional localisation information compared to a gamma image in isolation.

The depth of the radioisotope source below the anatomical surface can be calculated using two hybrid systems in a well-defined geometry, resulting in four images—two gamma and two optical. Using the known camera separation and the relationship between imaging distance and magnification allows the distance from the camera to the gamma source and from the camera to the surface of the object to be calculated. Combining these calculations allows an estimation of the depth below the surface of the gamma source.

To demonstrate proof of this concept, a single hybrid camera was translated horizontally by 20 mm to simulate a second camera system and the imaging process described above was repeated for each camera to phantom distance.

**Figure 6:**
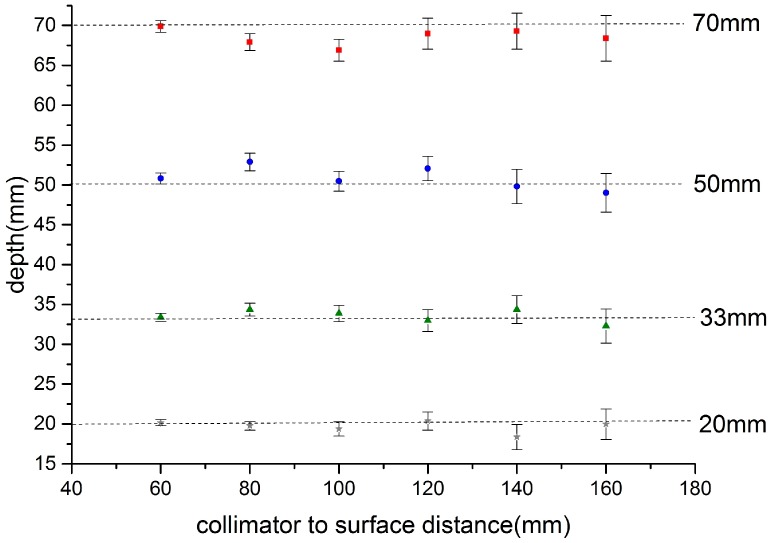
Relationship of calculated depth versus camera-to-phantom apex point for a source at the specified set positions below the apex of the breast phantom (Figure 4). Source position: squares 70 mm, circles 50 mm, triangles 33 mm, and crosses 20 mm.

Figure 6 shows the calculated depth of the source below the apex of the phantom over the range of source-to-phantom distances 60 mm to 160 mm. It is clear that the depth can be estimated consistently over this range. The main source of error is in the estimation of the source centre on each image which is, in part, related to the signal to noise of the images and centroid method employed. A future report will explore these issues and also describe the angular dependence of the depth estimation.

In this dual hybrid camera configuration four independent images are produced; two optical and two gamma which can be displayed as individual images, as a combined optical-gamma image (Figure 5), or as a single combined stereo image [20].

#### 3.1.2. Head and Neck Phantom Imaging

**Figure 7:**
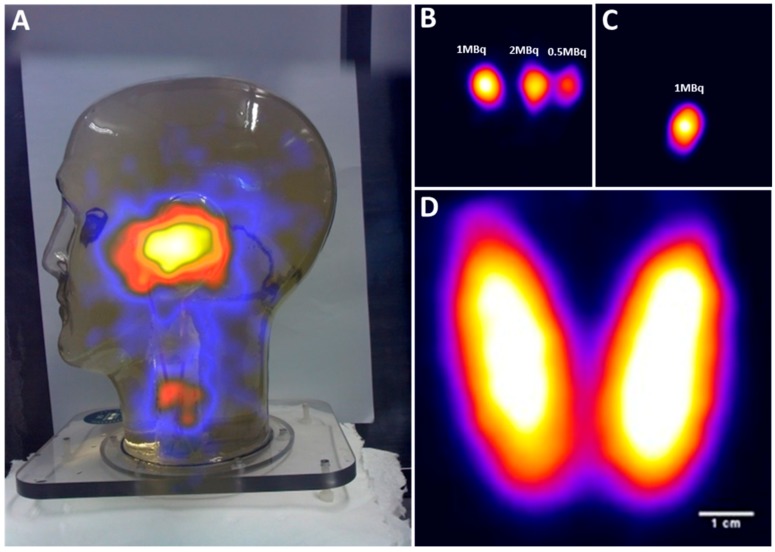
(**A**) A hybrid image of the head and neck phantom; (**B**) gamma image of three simulated parotid SLNs; (**C**) gamma image of simulated superficial cervical SLN; (**D**) gamma image of the thyroid phantom filled with 5 MBq of ^99m^Tc activity.

The internal components of the head and neck phantom were designed to simulate sentinel lymph nodes at various depths and height. Figure 7A–C shows the ability of the HGC to detect simulated SLNs in different regions in the head and neck.

In Figure 7A, the head and neck phantom were positioned 350 mm away from the HGC collimator face, and a hybrid gamma and optical image was produced (acquisition time was 8.5 mins). Four hot nodes have been positioned within the phantom. Three were placed level with the ear, with 35 mm separation between each node, to form a centrally-placed equilateral triangle if viewed from above. The fourth node was positioned in the neck.

The co-aligned configuration of the HGC enables an accurate localisation of the radioactive spots within the camera’s field of view. The three simulated parotid SLNs were detected within the 60 s acquisition time with the HGC fitted with a 1.0 mm diameter pinhole collimator and positioned 25 mm away from the phantom’s surface, as seen in Figure 7B. The middle parotid SLN containing 2 MBq of ^99m^Tc activity was positioned at a depth of 35 mm. The other two simulated nodes were located at a depth of 15 mm and they were filled with 1 MBq and 0.5 MBq of ^99m^Tc activity respectively as marked in Figure 7B.

Figure 7C shows a gamma image of a simulated superficial cervical SLN containing 1 MBq of ^99m^Tc activity (20 mm in depth); the HGC was 40 mm away from the phantom surface and the image was acquired in 60 s.

The life size thyroid phantom was used to simulate an adult thyroid gland in its proper anatomical position. The HGC was able to acquire a detailed gamma image while it was located 50 mm away (acquisition time is 5 min), with the thyroid phantom filled with 5 MBq of ^99m^Tc activity (Figure 7D). The thyroid phantom gamma imaging shows the HGC can be used for small organ imaging, which will enhance patient management and provides bedside nuclear imaging technology. Furthermore, the fused gamma-optical imaging provided by the HGC make it an effective tool to trace targeted SLNs during pre-, intra-, and post-operative nuclear imaging procedures.

#### 3.1.3. Fluorescent Hybrid Imaging

**Figure 8:**
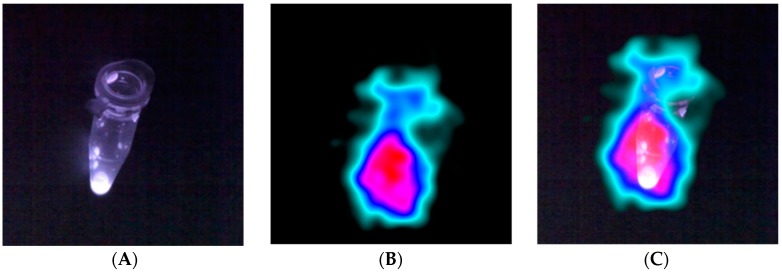
(**A**) Image showing fluorescence dye in a small Eppendorf; (**B**) the gamma ray image; (**C**) the fused fluorescence and gamma image.

Figure 8 shows the images resulting from the in vitro test using the hybrid gamma-fluorescent tracer. Figure 8A is the fluorescent NIR image, Figure 8B shows a gamma only image, and Figure 8C is the combined optical-gamma image. As can been seen from Figure 8, there was good registration between the NIR from the fluorescence dye and the gamma rays emitted from the ^111^In radionuclide tracer. This demonstrates the feasibility of the hybrid imaging concept, offering new potential for clinical applications.

### 3.2. In Vivo Imaging

The combination of gamma and optical imaging in a hybrid system will facilitate improvements in a number of medical and surgical procedures. The registration of the gamma image onto the optical image will allow the physicians to improve their assessment of radiopharmaceutical localisation during surgery and so speed up the process. As part of the development of the HGC, a number of volunteers who were having nuclear medicine examinations at Queen’s Medical Centre, Nottingham were imaged with the HGC following their regular medical tests.

#### 3.2.1. Clinical Imaging of the Thyroid Gland

**Figure 9:**
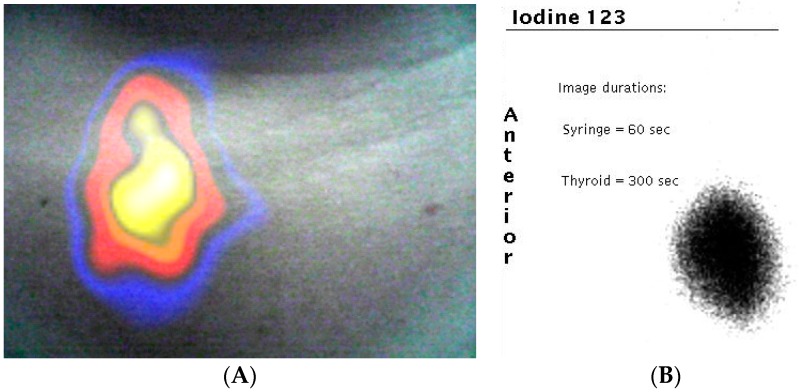
(**A**) Combined gamma and optical image of a patient’s thyroid during clinical investigation. The uptake of ^123^I is clearly seen in the right lobe of the patient’s thyroid (left side of image); (**B**) the standard clinical image taken by a large field of view gamma camera in the nuclear medicine clinic.

During a thyroid scintigraphy procedure, 20 MBq of ^123^I-NaI (159 keV) was administered intravenously 90 min prior to patient imaging. A number of gamma and optical images were taken at a range of patient to camera distances to ensure that the entire thyroid was within the FOV of the HGC. The gamma image was processed using a smoothing algorithm prior to being combined with the optical image. Although a reduction of spatial resolution may be noticed in the gamma image due to this process, it enhances the visual appearance when fused with its optical counterpart (Figure 9). The fused thyroid image (Figure 9) shows an active right lobe but no detectable activity present in the left lobe. This pattern of uptake was confirmed by imaging (Figure 9) using a standard Large Field of View (LFOV) gamma camera.

#### 3.2.2. Near-Infrared Preclinical Imaging

The modified fluorescent imaging component of the camera was tested for preclinical imaging in a licensed preclinical biomedical facility. An immunocompromised nude mouse with two orthotopic HCC-1954 tumour xenografts was injected with 32 MBq of ^111^In-DTPA-Trastuzumab-CW800 via a tail vein. Imaging was carried out after allowing sufficient time for accumulation of the injected tracer.

**Figure 10:**
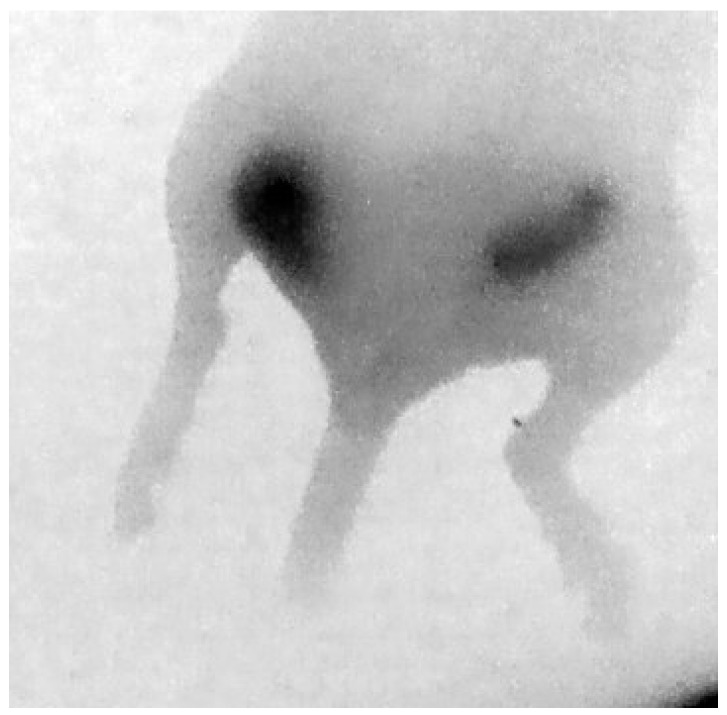
Posterior NIR fluorescence image of the hind section of a mouse showing uptake in two orthotropic tumours.

Figure 10 shows a fluorescent image of this mouse, taken with the modified HGC, where the two tumours are clearly visible.

## 4. Discussion and Conclusions

Imaging techniques are ubiquitous in medicine, and the use of intraoperative gamma imaging has the potential to further improve surgical outcomes. The concept of sentinel lymph node biopsy has been applied to assist in the identification of patients for complete lymph node dissection [21,22,23]. This technique is beneficial for the cancer staging process and to determine whether the primary tumour has spread to the surrounding lymphatic nodes. Such procedures are carried out over 2 million times annually throughout the world.

There may be many medical advantages of fusing the optical and gamma images by providing both anatomical and physiological information during surgical procedures. The HGC may provide practical benefits including bedside imaging for small targeted organs and tissues, such as thyroid gland and lacrimal ducts.

Using the HGC will provide the surgeon with fused optical and gamma images that can enhance the localisation of the targeted tissues in critical surgical situations. Furthermore, the HGC would help to minimise the chance of leaving abnormal tissues behind after the surgical procedure is complete.

The HGC has been utilised to produce multi-modal images from phantom simulations and patients undergoing clinical examinations. In addition, we have shown that the hybrid camera concept can be extended to produce stereoscopic imaging of both optical and gamma photons, which can be used to estimate the position (or depth) of radionuclide distribution within a patient or more generally an object.

There is currently no commercially available system that offers hybrid imaging for clinical use that combines fluorescence and gamma imaging into a single fused image. Development of such a system will have significant benefits for cancer diagnosis and treatment, especially in the case of head and neck cancers, and will be a unique offering in the healthcare arena which will make it extremely useful in-theatre for procedures such as sentinel lymph node biopsies. This communication describes for the first time a camera system that allows multiplexing of fluorescent and gamma probes during the same procedure, demonstrating the feasibility of hybrid gamma and fluorescence image guided surgery. Multiplexing will allow the surgeon to use a combination of probes to visualise different physiological and cellular markers, improving the overall sensitivity and specificity of feature detection. The HGC provides fused gamma and optical imaging that would offer a new tool to tackle existing and emerging medical challenges at the point of care and during intra-operative image guided surgery.

## References

[1] Bluemel C., Schnelzer A., Okur A., Ehlerding A., Paepke S., Scheidhauer K., Kiechle M. (2013). Freehand SPECT for image-guided sentinel lymph node biopsy in breast cancer. Eur. J. Nucl. Med. Mol. Imaging.

[2] Duch J. (2011). Portable gamma cameras: The real value of an additional view in the operating theatre. Eur. J. Nucl. Med. Mol. Imaging.

[3] Tsuchimochi M., Hayama K. (2013). Intraoperative gamma cameras for radioguided surgery: Technical characteristics, performance parameters, and clinical applications. Phys. Med..

[4] Del Sordo S., Abbe L., Caroli E., Mancini A.M., Zappettini A., Ubertini P. (2009). Progress in the development of CdTe and CdZnTe semiconductor Radiation Detectors for Astrophysical and Medical Applications. Sensors.

[5] Ferretti A., Chondrogiannis S., Marcolongo A., Rubello D. (2013). Phantom study of a new hand-held gamma-imaging probe for radio-guided surgery. Nucl. Med. Commun..

[6] Trotta C., Massari R., Palermo N., Scopinaro F., Soluri A. (2007). New high spatial resolution portable camera in medical imaging. Nucl. Instrum. Methods A.

[7] Siman W., Kappadath S.C. (2012). Performance characteristics of a new pixelated portable gamma camera. Med. Phys..

[8] Russo P., Curion A.S., Mettivier G., Esposito M., Aurilio M., Caracò C., Aloj L., Lastoria S. (2011). Evaluation of a CdTe semiconductor based compact gamma camera for sentinel lymph node imaging. Med. Phys..

[9] Olcott P.D., Habte F., Foudray A.M., Levin C.S. (2007). Performance Characterization of a Miniature, High Sensitivity Gamma Ray Camera. IEEE Trans. Nucl. Sci..

[10] Tanaka C., Fujii H., Shiotani A., Kitagawa Y., Nakamura K., Kubo A. (2005). Sentinel node imaging of laryngeal cancer using a portable gamma camera with CdTe semiconductor detectors. Clin. Nucl. Med..

[11] Abe A., Takahashi N., Lee J., Oka T., Shizukuishi K., Kikuchi T., Inoue T., Jimbo M., Ryuo H. (2003). Performance evaluation of a hand-held, semiconductor (CdZnTe)-based gamma camera. Eur. J. Nucl. Med. Mol. Imaging.

[12] Lees J.E., Bugby S.L., Bhatia B.S., Jambi L.K., Alqahtani M.S., McKnight W.R., Ng A.H., Perkins A.C. (2014). A Small Field of View Camera for Hybrid Gamma and Optical Imaging. J. Instrum..

[13] Valdés Olmos R.A., Vidal-Sicart S., Giammarile F., Zaknun J.J., Van Leeuwen F.W., Mariani G. (2014). The GOSTT concept and hybrid mixed/virtual/augmented reality environment radioguided surgery. Q. J. Nucl. Med. Mol. Imaging.

[14] Van den Berg N.S., Brouwer O.R., Klop W.M.C., Karakullukcu B., Zuur C.L., Tan I.B., Balm A.J.M., van den Brekel M.W.M., Valdés Olmos R.A., van Leeuwen F.W.B. (2012). Concomitant radio-and fluorescence-guided sentinel lymph node biopsy in squamous cell carcinoma of the oral cavity using ICG-99mTc-nanocolloid. Eur. J. Nucl. Med. Mol. Imaging.

[15] Brouwer O.R., Buckle T., Vermeeren L., Klop W.M.C., Balm A.J.M., van der Poel H.G., van Rhijn B.W., Horenblas S., Nieweg O.E., van Leeuwen F.W.B. (2012). Comparing the hybrid fluorescent–radioactive tracer indocyanine green–99mTc-nanocolloid with 99mTc-nanocolloid for sentinel node identification: A validation study using lymphoscintigraphy and SPECT/CT. J. Nucl. Med..

[16] Lees J.E., Bassford D.J., Blackshaw P.E., Perkins A.C. (2010). Design and use of mini-phantom for high resolution planar gamma cameras. Appl. Radiat. Isot..

[17] Alqahtani M.S., Lees J.E., Bugby S.L., Jambi L.K., Perkins A.C. (2015). Lymphoscintigraphic imaging study for quantitative evaluation of a small field of view (SFOV) gamma camera. J. Instrum..

[18] Ng A.H., Clay D., Blackshaw P.E., Bugby S.L., Morgan P.S., Lees J.E., Perkins A.C. (2015). Design and use of a sentinel node phantom for pre-surgical assessment of small field of view gamma cameras. Nucl. Med. Commun..

[19] Bugby S.L., Lees J.E., Ng A.H., Alqahtani M.S., Perkins A.C. (2016). Investigation of a SFOV hybrid gamma camera for thyroid imaging. Phys. Med..

[20] Lees J.E., Bugby S.L., Bark A.P., Bassford D.J., Blackshaw P.E., Perkins A.C. (2013). A Hybrid Camera for locating gamma sources in the environment. J. Instrum..

[21] Salvador S., Bekaert V., Mathelin C., Guyonnet J.L., Huss D. (2007). An operative gamma camera for sentinel lymph node procedure in case of breast cancer. J. Instrum..

[22] Koops H.S., Doting M.H.E., de Vries J., Tiebosch A.T.M.G., Plukker J.T., Hoekstra H.J., Piers D.A. (1999). Sentinel node biopsy as a surgical staging method for solid cancers. Radiother. Oncol..

[23] Giammarile F., Alazraki N., Aarsvold J.N., Audisio R.A., Glass E., Grant S.F., Kunikowska J., Leidenius M., Moncayo V.M., Uren R.F. (2013). The EANM and SNMMI practice guideline for lymphoscintigraphy and sentinel node localization in breast cancer. Eur. J. Nucl. Med. Mol. Imaging.

